# Supplementary material for: Small-scale displacement fluctuations of vesicles in fibroblasts
Source: Sci Rep. 2018 Sep 5;8:13294. doi: 10.1038/s41598-018-31656-3 (PMC6125338; doi:10.1038/s41598-018-31656-3)
Supplement: Supplementary file 1 — Supplementary Material [file 41598_2018_31656_MOESM1_ESM.pdf]

Small-scale displacement fluctuations of vesicles in fibroblasts

Danielle Posey[1], Paris Blaisdell-Pijuan[2], Samantha K. Knoll[3], Taher A. Saif[3], and Wylie W. Ahmed[2,*]

[1]Department of Biological Sciences, California State University, Fullerton, CA
[2]Department of Physics, California State University, Fullerton, CA [3]Department of Mechanical Science and Engineering, University of Illinois, Urbana, IL [*]Corresponding author (wahmed@fullerton.edu)

Supplementary Material

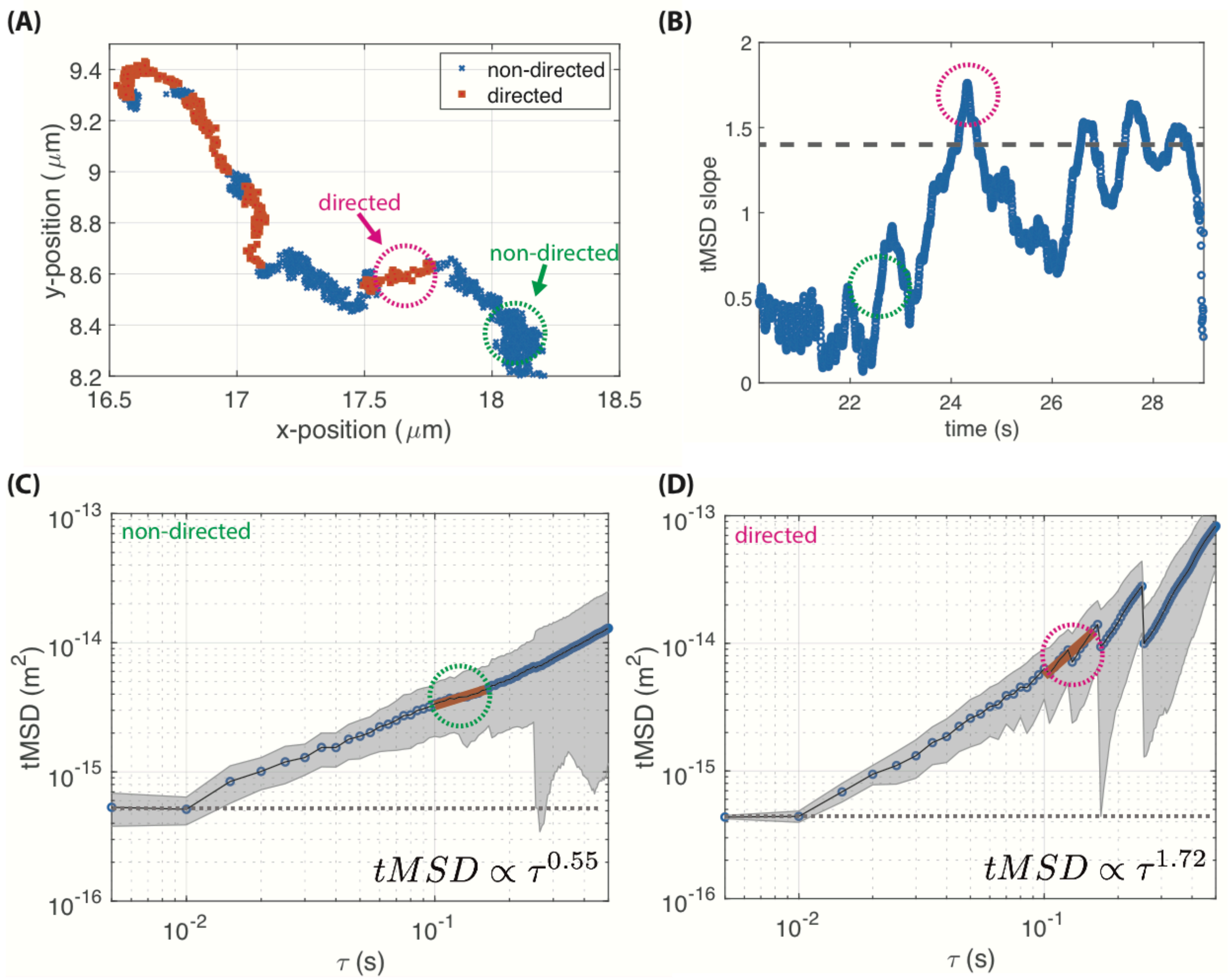

Figure S1: (A) A representative trajectory of a vesicle indicating directed (squares) and non-directed (x) motion. The circled regions for directed (magenta) and non-directed (green) correspond to the circled data in B,C, and D. (B) The power-law of the tMSD analysis plotted as a function of time. (C) A representative tMSD of a vesicle undergoing non-directed motion with a sub-diffusive power-law of 0.55. The horizontal dashed line indicates the tracking uncertainty (~23 nm). (D) A representative tMSD of a vesicle undergoing directed motion with a super-diffusive power-law of 1.72. The horizontal dashed line indicates the tracking uncertainty (~21 nm). As evidenced by (C) and (D), the tMSD analysis is well-above the tracking uncertainty. (shaded gray region indicates standard deviation)

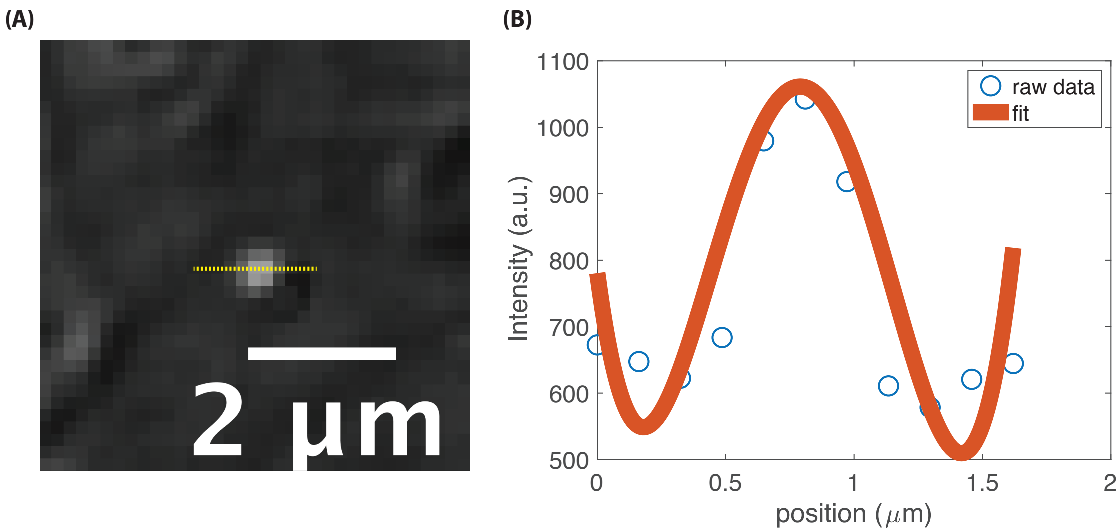

Figure S2: (A) A representative differential interference contrast (DIC) microscopy image of a vesicle in a fibroblast. Horizontal dashed yellow line indicates the region of intensity measurements for fitting. (B) Example of raw intensity data (circles) with a polynomial fitting with Gaussian weight (solid line) indicating the algorithm developed by Rogers et al. Physical Biology 2007 is able to fit particles in DIC images.
